# Bifocal flat lens with different imaging characteristics for a dual-sensor imaging system

**DOI:** 10.1038/s41598-022-22103-5

**Published:** 2022-11-08

**Authors:** Yin Zhou, Feng-Lin Kuang, Rui Li, Lei Li

**Affiliations:** School of Electronics and Information Engineering, Sichuan University, Chengdu, 610065 China

**Keywords:** Engineering, Optics and photonics, Physics

## Abstract

Wide field of view (FOV) images and magnified images can be taken simultaneously by dual-sensor imaging systems. Here, we propose an approach for creating a bifocal flat lens with different imaging characteristics of its two foci, which makes dual-sensor imaging systems more integrated and miniaturized. That is, two special parts of two different conventional ZP are extracted and then combine the two elements in a specific way. So that there are two foci with different characteristics along the optical axis, one is long focus with higher resolution, the other is short focus with long depth of focus (DOF). Under the proposed approach, a thin and light bifocal diffractive lens (BDL) with thickness of 0.6 μm is developed. The long and short focal lengths of the BDL are ~ 81 mm and ~ 27 mm, respectively, with a diameter of 6 mm. We experimentally demonstrate that the long focus of the BDL is capable of taking high-resolution magnified images, and its resolution is up to 21.90″. The short focus is able to take wide FOV with long DOF images, and two objects spread 2880 mm apart can be imaged clearly. The experiment results demonstrate that all of these metrics are better than those of a conventional refractive lens.

## Introduction

In imaging system, magnification imaging and wide field of view (FOV) imaging are very important in order to get more information^1^. Applications that require magnification imaging and wide FOV imaging range from surveillance^2^ and medicine^3–5^ to artificial intelligence^6,7^. Since with just one focus in a conventional imaging system, it requires zoom to take magnified images and wide FOV images, which means magnified images and wide FOV images cannot be taken by the conventional zoom imaging systems simultaneously. In addition, zoom imaging systems are very thick and bulky, and the zoom process is time-consuming as well as hard to balance wide FOV and high resolution well. To solve these problems, many approaches have been proposed, such as lens-array imaging^8–14^ and foveated imaging^15–18^ and dual-camera system^19^ and dual-sensor imaging system^20^. However, the drawbacks of these approaches are very bulky and complicated due to more than one lens, which limits their application in wide fields.

For lightweight and integration and miniaturization purpose, flat lenses with two foci provide a solution for dual-sensor imaging system, which means the long focus enables high-resolution magnification imaging, while short focus enables wide FOV with long DOF imaging, simultaneously. Flat lenses include metalenses and diffractive optical elements (DOEs), and both of them are able to manipulate the phase of light freely. Furthermore, the manipulation of the polarization can be achieved by metalenses^21^. Metalenses are novel optical devices proposed in recent years^22,23^, and it can achieve the designed wavefront by arranging these units carefully, including achromatism^24,25^ and polarization imaging^26^. Nonetheless, this is a phase control problem in this paper instead of polarization control, and it is a great challenge to design and fabricate a metalens with larger constituent features compared to DOEs^27^. Therefore, the DOEs are more suitable for meeting these requirements, and it is much simpler to manufacture thanks to the development of photolithography technology, making them accessible to low-cost, large-area volume manufacturing. There are two common approaches to designing a multifocal DOE, including optimizing given intensity distribution^28–31^ and designing diffraction orders^32–34^. For the first approach, that is, some algorithms are used for optimizing optical elements to form a given intensity distribution along the optical axis. For the second approach, it is a binarization of an ordinary lens, that is, additional diffraction orders appear along the optical axis. Both of them have been studied for a long time and are well known. However, the drawback of the first approach is that they require optimization algorithm to optimize given intensity distribution along the optical axis, which is time-consuming and difficult to manufacture large multifocal DOEs. The drawback of the second approach is that a series of extra focal spots appear on the optical axis, reducing the quality of primary focus imaging we need. Besides, it is difficult to achieve the expected focal characteristics along the optical axis by binarization of an ordinary lens to produce additional foci.

To solve these problems, we propose an approach for creating a bifocal flat lens with different imaging characteristics of its two foci, especially suitable for application scenario here. The bifocal flat lens is actually a two-area diffractive element based on the Fresnel zone plate (FZP). The FZP has been used in many applications, such as optical imaging and X-ray imaging. However, because of the large chromatic aberration of the FZP due to its work principle, it is usually employed in single-wavelength imaging instead of broadband imaging, which could be achieved by multilevel diffractive lenses (MDL)^35,36^. Hence, the proposed bifocal flat lens is suitable employed in single-wavelength imaging. Each area corresponds to part of a zone plate with a specific focal length. Special imaging characteristics of the focal spots can be achieved through a specific combination of these two elements. Therefore, the proposed approach of creating a bifocal flat lens enables large size fabrication and high-quality imaging by avoiding algorithm optimization or generating a series of extra focal spots.

Here we propose an approach to design a thin and light bifocal diffractive lens (BDL) which can directly take a high-resolution magnified image and a wide FOV with long depth of focus (DOF) image simultaneously through its two foci. By designing two high intensity focal spots along the optical axis of the BDL, two high-quality images can be taken at the same time, including a magnified image and a wide FOV image. Moreover, the long DOF characteristic of the short focus is achieved via the proposed designing approach of bifocal flat lens, while the high-resolution characteristic of the long focus is achieved as well. Therefore, the BDL is able to take a high-resolution magnified image and a wide FOV with long DOF image simultaneously through the two specific designed foci, which makes the dual-sensor imaging system more integrated and miniaturized.

## Design

**Figure. 1:**
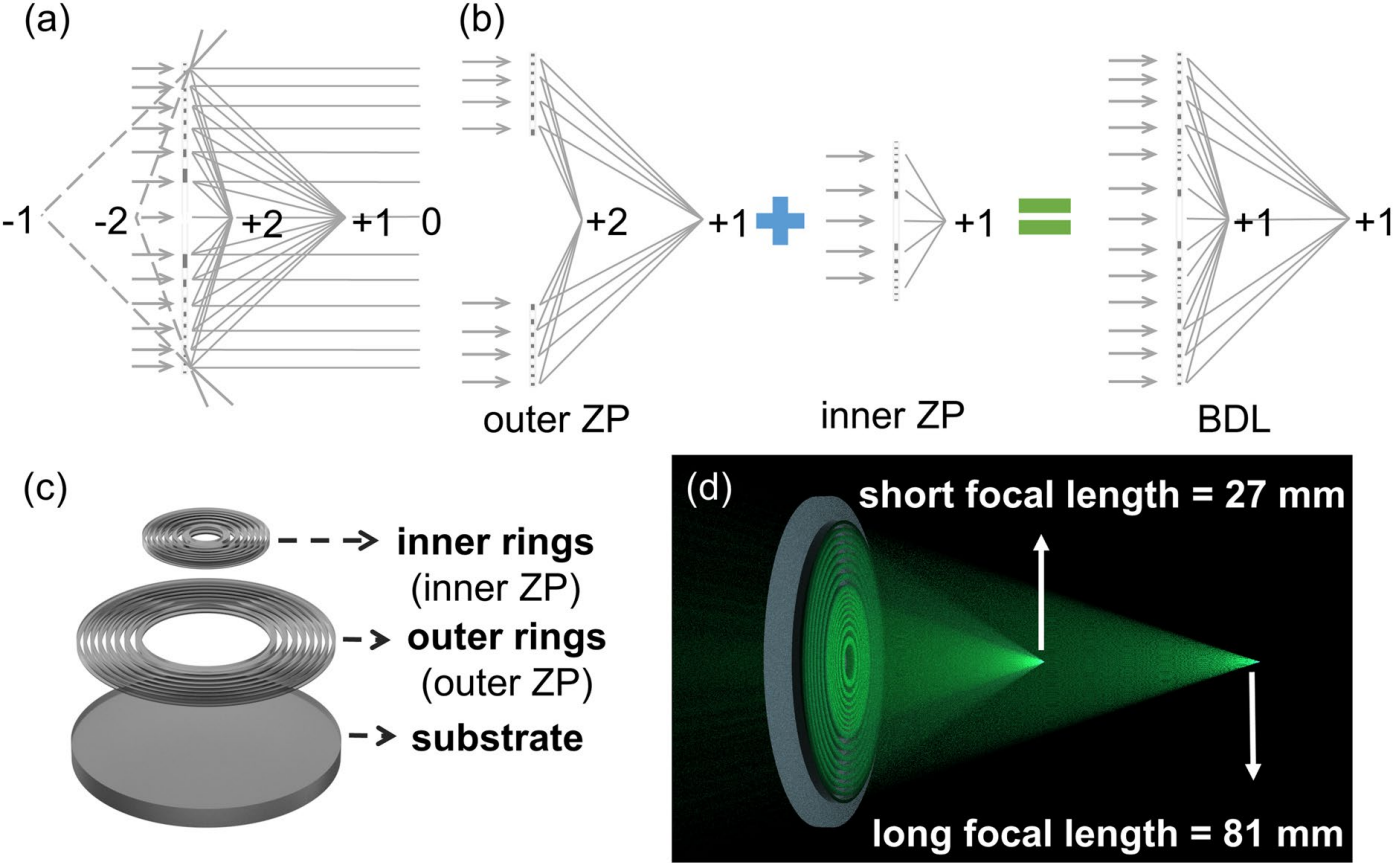
(**a**) Diffraction-order focuses of the FZP. (**b**) Schematic diagram of the design process of the BDL diffraction orders. (**c**) Schematic of the structure of the BDL. (**d**) Diagram of the working principle of the BDL.

Our design is inspired by a conventional multifocal DOE, which is Fresnel zone plate (FZP)^37^. Figure 1a depicts the diffraction-order focuses of the FZP. When a parallel beam of light illuminates on the FZP, the + 1 and + 2 diffraction-order focuses and − 1 and − 2 diffraction-order focuses are on either side of the lens, and zero diffraction order plays a role as background noise. The imaging quality of first-order focal spot of the FZP is excellent due to high intensity focusing, while the imaging qualities of other foci become poor. Obviously, focusing characteristics which are needed here can be achieved by combination of these diffraction orders, as shown in Fig. 1b. From the figure, it is clear that the problem of combination of these diffraction orders can be solved via combining two conventional ZPs. One conventional ZP is a long focal length ZP for the first-order with hollow inside, named outer ZP. The other one is a short focal length ZP for the first-order, named inner ZP. The short focal length is equal to 1/3 of the long focal length, so that the second-order focus of the outer ZP coincides with the first-order focus of the inner ZP. By this particular combination, there are two high-intensity (first-order) focal spots along the optical axis, which means two different pictures with high quality can be taken by the two foci. One is a magnified image taken by the long focus, and the other one is a wide FOV image taken by the short focus. Moreover, through this specific combination, the long focus can take higher resolution image by using medium–high spatial frequency component, and the short focus can take long DOF image via generating a high intensity focal spot. Although there have been many examples of enhance DOF^38–41^, the approach we propose can directly enhance the DOF without requiring extensive post-processing or multiple elements to be quite thick. Thus, the composite lens which is named bifocal diffractive lens (BDL) can directly take a high-resolution magnified image and a wide FOV with long DOF image simultaneously through its two specific designed foci.

Figure 1c depicts the structure of the proposed BDL. The outer rings represent the component of the outer ZP, and its first-order focal length is the long focal length. The inner rings represent the component of the inner ZP, and its first-order focal length is the short focal length. Figure 1d shows the diagram of the working principle of the BDL.

In mathematical terms, since the BDL is designed based on the conventional ZP, the designed radii *r*_*inner*_ and *r*_*outer*_ for the inner rings and outer rings are calculated as follows:

1 $$ r_{inner}^{2} = S \cdot \lambda f, $$

2 $$ r_{outer}^{2} = S \cdot 3\lambda f, $$

where *f* is the first-order focal length (short focal length) of inner rings, *λ* is the designed operating wavelength, and *S* is the number of rings. Transform Eqs. (1) and (2) into the transmittance function *q*_*inner*_(*ζ*) and *q*_*outer*_(*ξ*), respectively. It should be noted that the BDL is a combination of these two types of rings, so the transmittance function *q*(*ς*) of the BDL can be expressed as

3 $$ q_{BDL} (\varsigma ) = q_{inner} (\zeta ) + q_{outer} (\xi ). $$

Finally, using the Fresnel approximation, the axial irradiance can be calculated by the paraxial diffraction as^42^

4 $$ I(u) = 4\pi^{2} u^{2} \left| {\int_{0}^{1} {q_{BDL} } (\varsigma )\exp \left( { - i2\pi u\varsigma } \right)d\varsigma } \right|^{2} , $$

where *u* = *a*^2^/(*2λz*) is the reduced axial coordinate, *a* is the radius of the BDL. *λ* and *z* are the designed operating wavelength and axial distance, respectively.

In this paper, the long and short focal lengths of our designed BDL are 81 mm and 27 mm, respectively. The diameter of the inner ZP is 3 mm, and the width of the outer ZP is 1.5 mm. That is, the inner ZP and outer ZP each occupy half of the whole radius of the designed BDL. Besides, the designed operating wavelength is 532 nm.

## Focusing properties

**Figure. 2:**
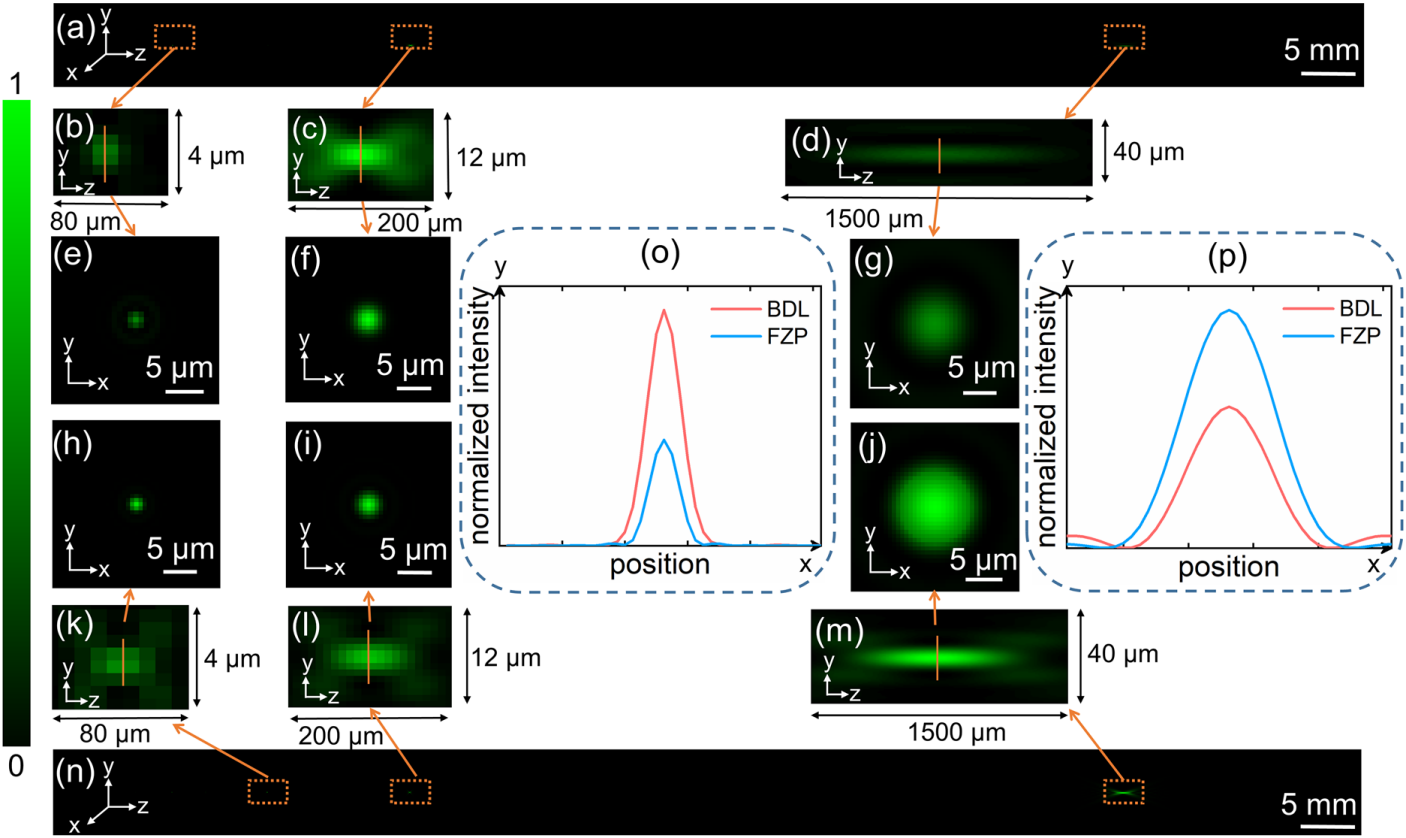
Focusing properties of the BDL and FZP. (**a**) Axial intensity distribution of the BDL. (**b**) Axial intensity distribution of the third focus of the BDL. (**c**,**d**) Axial intensity distribution of the short and long foci of the BDL. (**e**–**g**) The intensities of corresponding focal spots of the BDL. (**h**–**j**) The intensities of corresponding focal spots of the FZP. (**k**) Axial intensity distribution of the third focus of the FZP. (**l**,**m**) Axial intensity distribution of the short and long foci of the FZP. (**n**) Axial intensity distribution of the FZP. (**o**) Comparison of the short focus of the BDL and FZP. (**p**) Comparison of the long focus of the BDL and FZP.

In order to analyze focusing characteristics of the BDL, the axial diffraction simulation of the BDL is done based on Eq. (4). The results are shown in Fig. 2. For comparison, the conventional diffractive optical element FZP is done axial diffraction simulation as well. The complete axial intensity distributions of the BDL and FZP are shown in Fig. 2a,n. From the figures, it is apparent that there are three distinct focal spots of both lenses, and all of them are extracted to make them see clearly and easy comparison, as shown in Fig. 2b–d and k–m. At the same time, their corresponding focal spots are extracted as well, as shown in Fig. 2e–j. Figure 2b,k are axial intensity distributions of the third focal spots of the BDL and FZP, respectively, and they are the redundant focuses and as background noise for other foci. It can be seen that the diameter and intensity of the third focal spot of the BDL is smaller than that of FZP, which means the effect on other foci is less than FZP.

Figure 2c,l are axial intensity distributions of short focal spots of the BDL and FZP, respectively. The comparison result of them is shown in Fig. 2o. It is clear that the length and intensity of the short focal spot of the BDL is bigger than that of FZP, which means a wide FOV with long DOF image can be captured by the short focal spot of the BDL.

Figure 2g,j are long focal spots of the BDL and FZP, respectively, and both of them are compared in Fig. 2p. It is obvious that the diameter of the long focal spot decreases, which means the long focal spot of the BDL achieves better focusing performance and higher resolution, although the intensity of the long focus decreases as well. Thus, a high-resolution magnified image can be taken by the long focus of the BDL. Notably, the efficiencies of the BDL at *f* = 81 mm and *f* = 27 mm are 13.11% and 6.45%, respectively. For the FZP at *f* = 81 mm and *f* = 27 mm are 18.78% and 2.21%, respectively.

## Experiment

### Fabrication

**Figure. 3:**
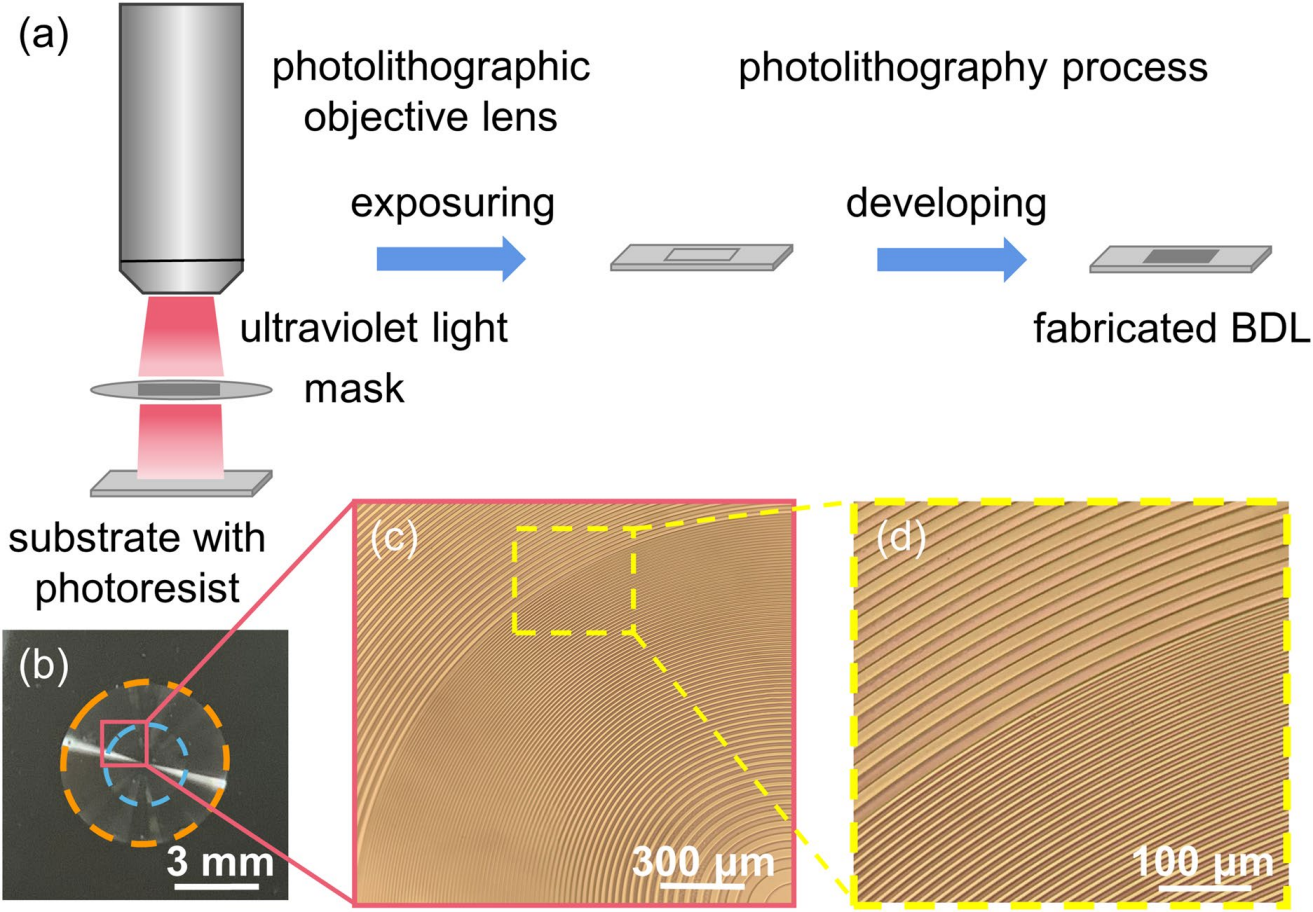
(**a**) Flow chart of the photolithographic process. (**b**) The fabricated BDL. (**c**,**d**) Micrographs of the fabricated BDL.

The designed BDL is fabricated by using photolithography on a negative photoresist. Figure 3a shows the photolithography process. A layer of negative photoresist (SU-8, Microchem) is over the substrate of glass (silicon dioxide). By placing the designed mask between the photolithographic objective lens and the substrate coated with negative photoresist, ultraviolet light solidifies the exposed photoresist with turning on the light source of the lithography machine. Then with developing, the final whole BDL is fabricated, as shown in Fig. 3b. Figure 3c,d are micrographs of the fabricated BDL. The whole size of the substrate is 12 mm (length) × 12 mm (width). The diameter of the fabricated BDL is 6 mm. Furthermore, the height of these rings is 0.6 μm, and the width of the narrowest ring is 3 μm. As designed, the diameter of the inner ZP of the fabricated BDL is 3 mm, and the width of the outer ZP is 1.5 mm. The designed operating wavelength is 532 nm.

### Experimental setup

**Figure. 4:**
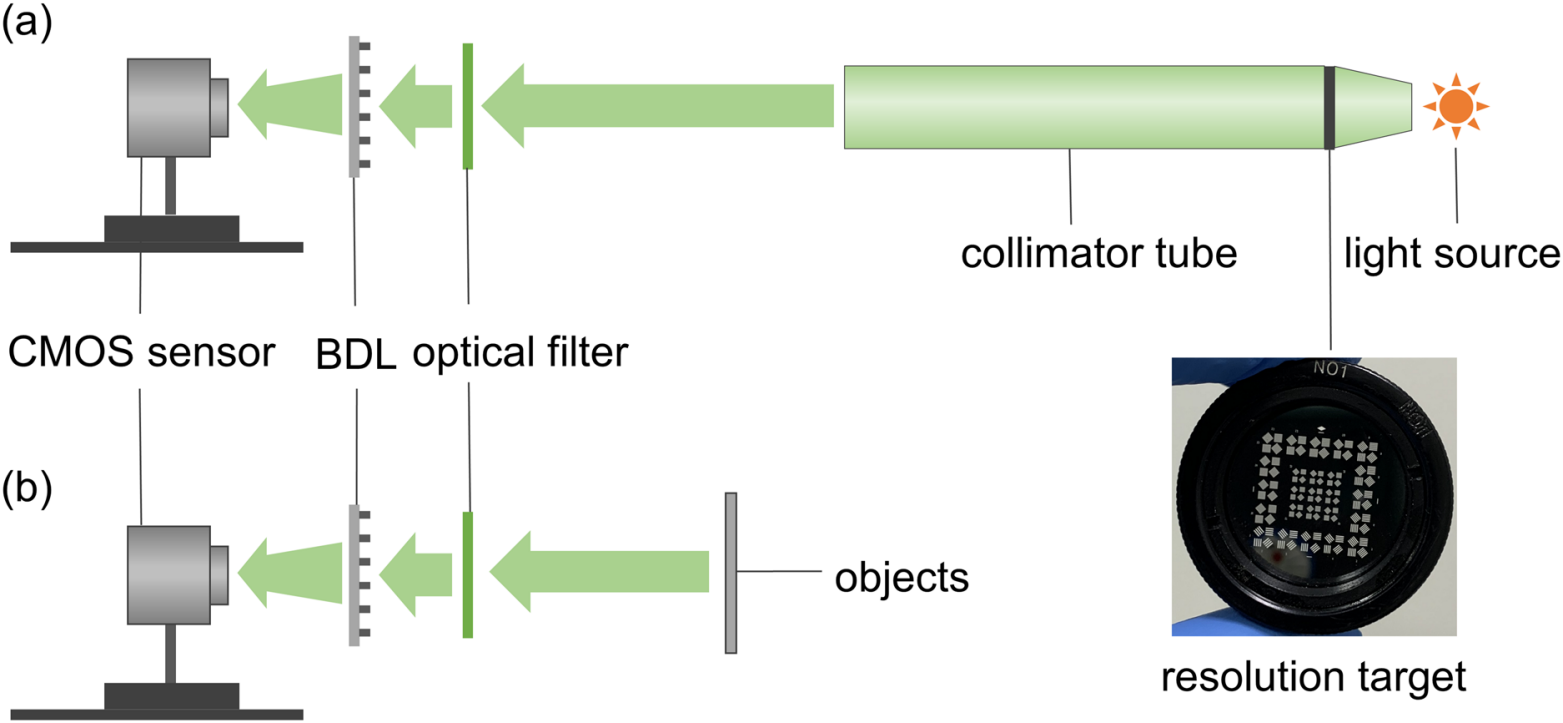
Diagram of experimental setups. (**a**) Schematic diagram of the resolution testing experiment setup. (**b**) Schematic diagram of the actual objects imaging experiment setup.

In order to demonstrate the imaging characteristics of the long and short focus of the BDL, two kinds of experiments with the long and short focus of the BDL are done, including resolution target testing and actual objects imaging testing. The schematic diagrams of corresponding two kinds of experiment setups are shown in Fig. 4a,b. For resolution target testing, a collimator tube (FPG-7, Huazhong Precision Instruments Co., Ltd., China) with a white light source and an optical filter (GCC-202105, Daheng Optics Co., Ltd., China) are used in the resolution target testing experiment. The BDL is located in between the collimator tube and the CMOS sensor (FT-GS500C, Fangte Technology Co., Ltd., China). The size of the CMOS sensor is 1/2.5″, and the pixel size and resolution are 2.2 µm × 2.2 µm and 2592 × 1944 pixels, respectively. The images are recorded by axial moving the position of the CMOS sensor. Notably, the distances between the BDL and CMOS sensor for the measurements are around 27 mm and 81 mm, which are approximately equal to the corresponding focal lengths of the long focus and the short focus of the BDL, respectively, because the measurements are infinite distance imaging through the collimator tube.

In actual objects imaging testing, the same optical filter and COMS sensor are used as well. Notably, the imaged object is changed from a resolution target to actual objects. Similarly, the images are recorded by axial moving the position of the CMOS sensor. In all objects imaging experiments, the objective distances are deliberately designed at a further long distance compared to the focal lengths of the BDL. Due to this, the image distance (the distance between the BDL and CMOS sensor) *v* = 1/(1/*f*—1/*u*) ≈ 1/1/*f* = *f*. Therefore, it can be viewed as infinite distance imaging, which means the distances between the BDL and CMOS sensor approximately equal to corresponding focal lengths of the short and long focus (27 mm and 81 mm), respectively.

### Characterization of long focus

**Figure. 5:**
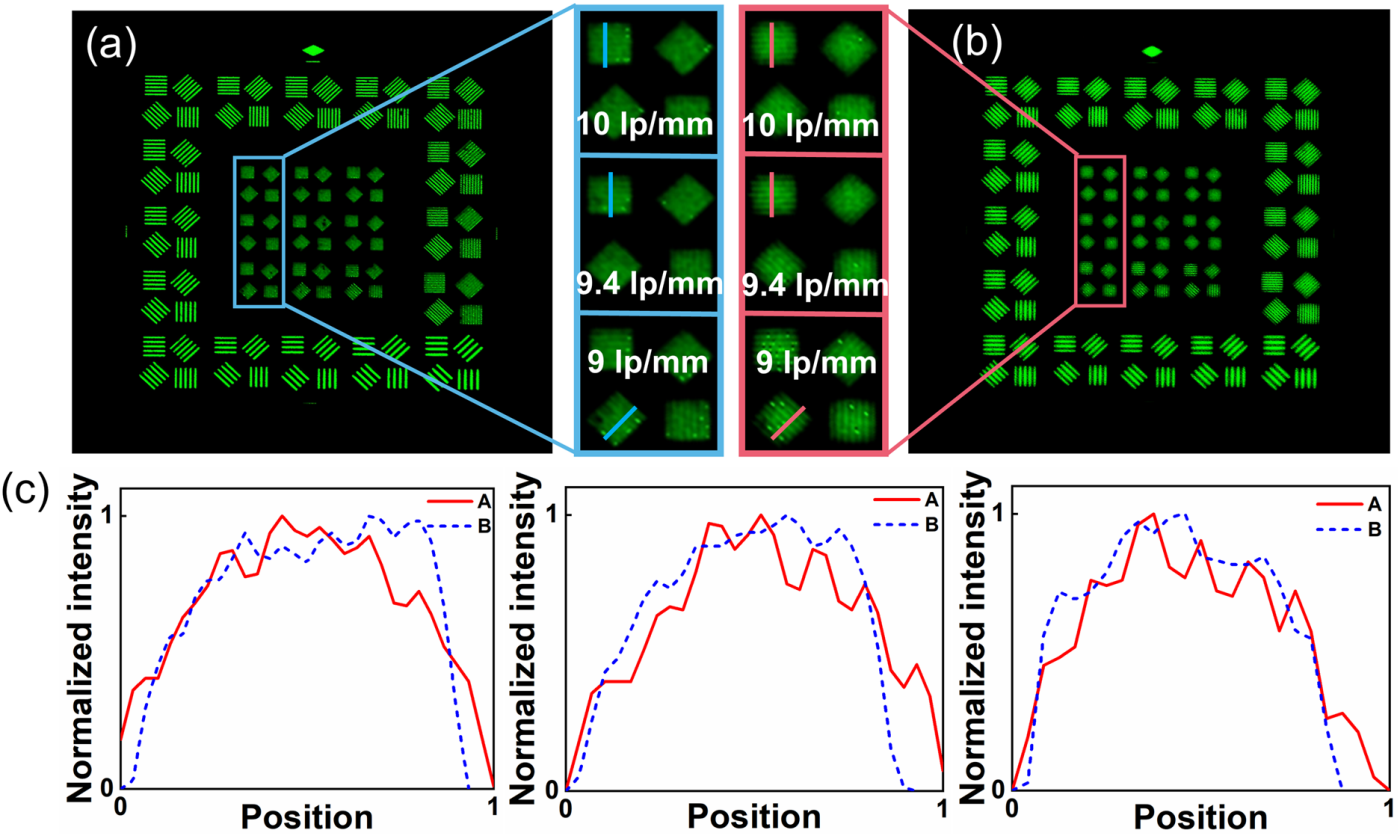
Resolution testing of the long focus. (**a**) Resolution image taken by the refractive lens. (**b**) Resolution image taken by the BDL. (**c**) Grayscale visualization curves of the small target images (A: BDL, B: Refractive Lens).

High resolution characteristic of the long focus of the BDL is demonstrated. In order to show the resolution performance of the long focus, the BDL and the conventional refractive lens (the same focal length and diameter as the BDL) are measured together for comparison. The resolution target is used for testing the resolution of the long focus of the BDL and FZP, as shown in Fig. 4a. The measured results of the conventional refractive lens and BDL are shown in Fig. 5a,b, respectively. For the 9 lp/mm resolution target, it is clear that the lines of the resolution test target can be distinguished by the BDL, whereas the refractive lens cannot distinguish these lines. To make the result more evident, the grayscale visualization curves of smaller target images are plotted, as shown in Fig. 5c. Compared with the conventional refractive lens, the curve of the BDL fluctuates even more sharply. Although the image appears sharp and uneven due to the loss of low spatial frequency, this does not affect its characteristic of high-resolution details magnified imaging. Through the experiment, the resolution of the conventional refractive lens is 24.60″, while the resolution of the long focus of the BDL is up to 21.90″, which means the BDL has higher resolution performance. In brief, the results indicate that the long focus of the BDL achieves higher-resolution imaging by using medium–high spatial frequency.

### Characterization of short focus

**Figure. 6:**
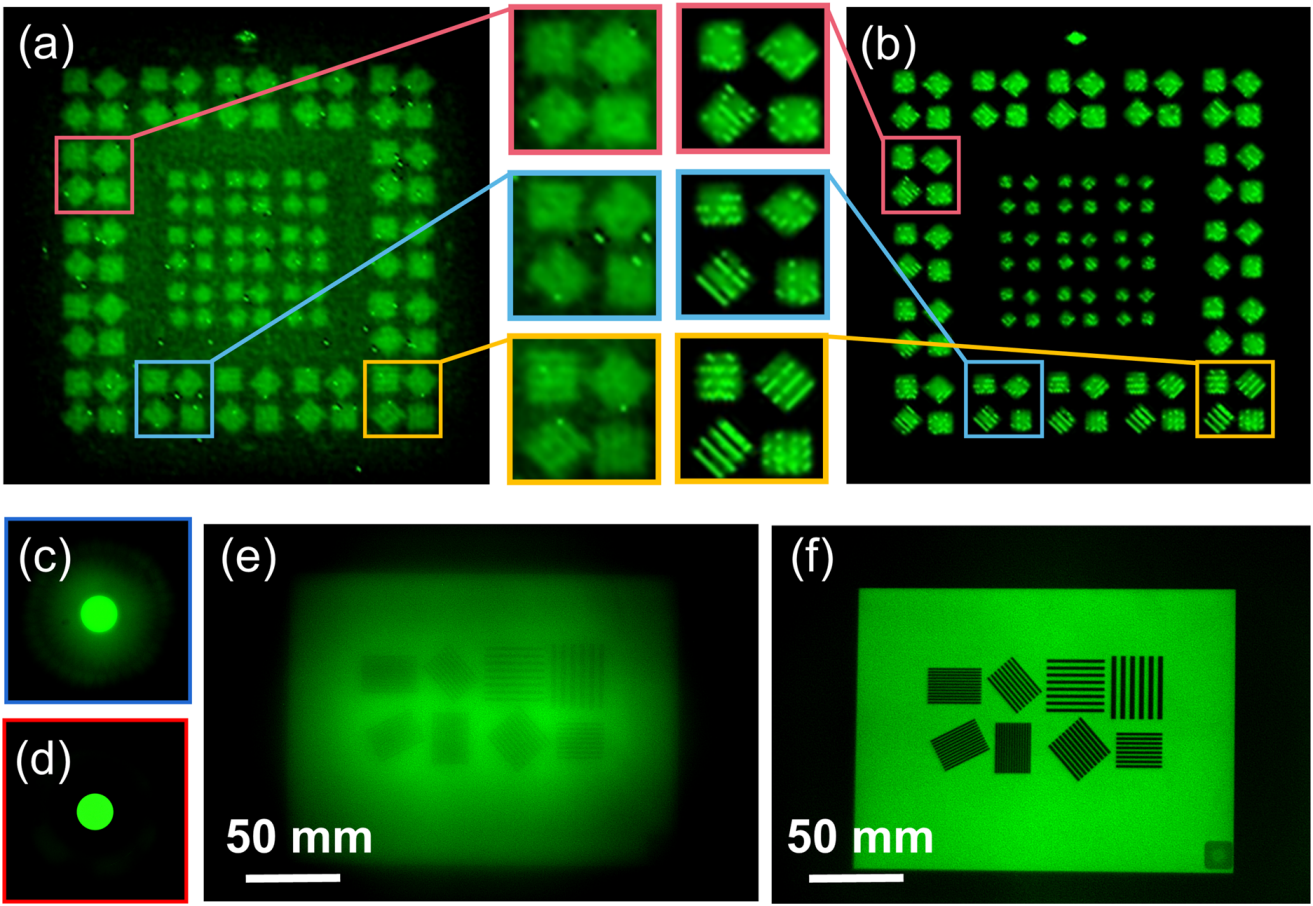
Imaging quality testing of the short focus. (**a**) Resolution image taken by the FZP. (**b**) Resolution image taken by the BDL. (**c**) Focal spot at short focal plane of the FZP. (**d**) Focal spot at short focal plane of the BDL. (**e**) Actual object image taken by FZP. (**f**) Actual object image taken by the BDL.

In order to demonstrate the high-quality imaging of the short focus of the BDL, the resolution of the short focus of the BDL is measured by imaging the same resolution target. The proposed BDL was designed based on the FZP, and the short focus (second-order focus) of the FZP could not imaging as mentioned before. Therefore, the specific resolution values were listed to show its quantized improvement. To make the results obvious, we compare the short focus of the BDL with corresponding short focus of FZP (the same focal length and diameter as the BDL). The measured results are shown in Fig. 6a,b. It is apparent that the image quality of the short focus has been greatly improved in the BDL. According to the experimental results, the resolution of the short focus of the FZP is just 1′ 06″, but the resolution of the short focus of the BDL is up to 44.14″. The focal spots of the FZP and BDL at short focal planes are also recorded in Fig. 6c,d for comparison, and it is clear that the BDL focuses better than FZP. In short focus imaging, although some details of the image are lost due to the loss of high spatial frequency, the imaging of objects in the wide FOV is not significantly affected.

In addition, actual object imaging experiment is also done to show its actual imaging performance. In the experiment, one iPad (iPad-2018, APPLE Co., Ltd., USA) is used as the object, and the screen size of the iPad is 9.7 inches. There are eight patterns on the iPad screen, and each pattern is made up of lines of different thickness. The distance between the iPad and the BDL is 2150 mm. The measured results of the FZP and BDL are shown in Fig. 6e,f. Obviously, the BDL has better imaging capability than FZP, and it can distinguish more lines.

**Figure. 7:**
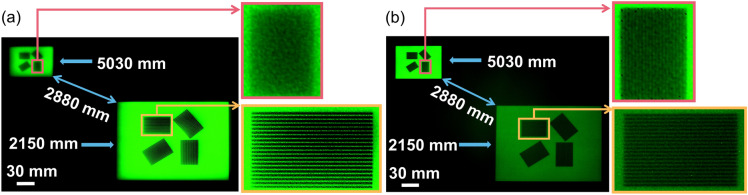
Long DOF imaging through the short focus. (**a**) Image taken by the refractive lens. (**b**) Image taken by the BDL. The distance between two objects is 2880 mm.

The depth of field (DOF) of the short focus of the BDL is measured. The conventional refractive lens is used as a comparison to make the results more intuitive. Two iPads at different positions are used as objects to measure. There are four patterns on the screen, and all of them consist of lines of the same thickness. The width of the lines on the screen of the iPad is 1.0 mm, and the distance between the lines is 0.9 mm. One of the iPads is placed 2150 mm in front of the BDL, and another iPad is placed 5030 mm in front of the BDL. Thus, the distance between these two iPads is 2880 mm. The measured results of the conventional refractive lens and BDL are shown in Fig. 7a,b, respectively. It shows that both the BDL and the refractive lens can take clear pictures of the closer iPad. However, the BDL can take clear image of the far iPad as well, but the refractive lens cannot. And the serious distortion is observed for the conventional refractive lens, whereas obvious distortion for the BDL cannot be observed. As expected, the experimental result shows that the BDL can take a long DOF image without obvious distortion through its short focus. The length of the DOF for short focus is ~ 200 μm according to the experiment result.

### Application in a dual-sensor imaging system

**Figure. 8:**
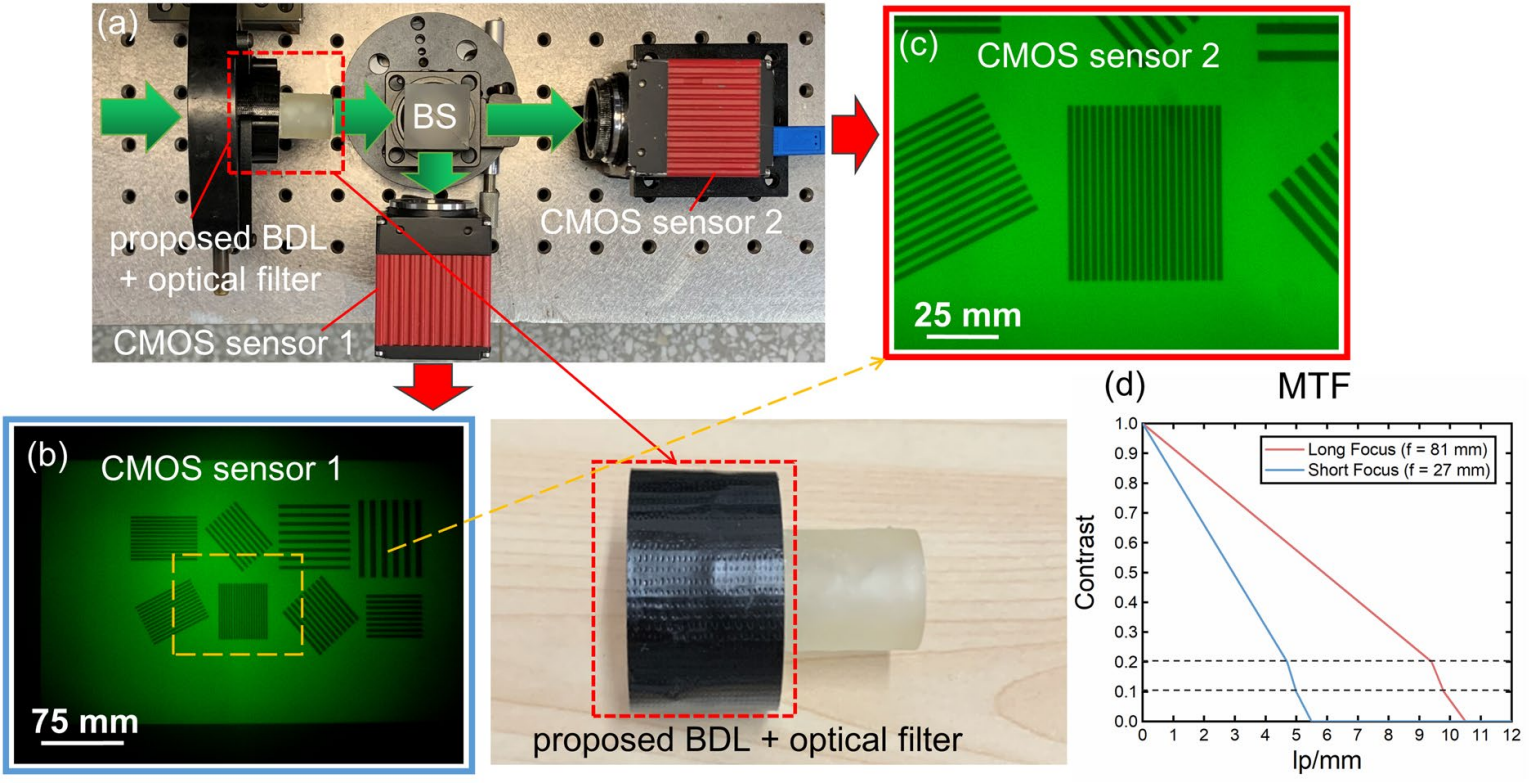
A dual-sensor imaging system employing the BDL. (**a**) Setup of the imaging system. (**b**) Wide FOV image taken by CMOS sensor 1. (**c**) High-resolution magnified image taken by CMOS sensor 2. (**d**) The MTF of the long and short focal spots of the BDL.

The BDL is suitable to be used in a dual-sensor imaging system. The experiment setup is shown in Fig. 8a, and the imaging system consists of the BDL and the optical filter. A display screen (P2415Q, DELL Co., Ltd., USA) is used as an object to measure. The size of the screen is 530 mm × 300 mm. There are eight patterns on the screen, and all of them consist of lines with different thicknesses. The width of the narrowest lines is 1.8 mm, and the shortest distance between these lines is 0.65 mm. All the patterns on the screen are recorded by the BDL through its short focus, as shown in Fig. 8b, which demonstrates that a wide FOV image can be recorded by the short focus of the BDL. Figure 8c shows a magnified pattern recorded by the long focus of the BDL, and these lines can be clearly distinguished. The result indicates that the detailed image of a part of a wide FOV can be recorded through the long focus of the BDL. It is obvious that the two images with different characteristics are taken by the BDL, simultaneously. The angle of the FOV for short focus is ~ 12° according to the experimental results. Notably, wide FOV (~ 12°) is just compared to the long focus of the BDL (~ 4°). The MTF of the long (*f* = 81 mm) and short focal spots (*f* = 27 mm) of the BDL were calculated by the resolution targets imaging, and plotted in one chart, as shown in Fig. 8d.

From the experiment, the results demonstrate that a wide FOV with long DOF image can be recorded by CMOS sensor 1, and a magnified image with high resolution can be recorded by CMOS sensor 2. Thus, the imaging system employing the BDL enables simultaneous high-resolution magnification imaging and wide FOV with long DOF imaging, which is possible to make a dual-sensor imaging system more integrated and miniaturized, thereby reducing weight, cost and associated complexity.

## Conclusion

In summary, we propose an approach to develop a bifocal diffractive lens and demonstrate a thin and light bifocal diffraction lens with different imaging characteristics of its two foci. The BDL is able to directly take two high-quality images with different characteristics through its specific designed two foci at the same time. One is the high-resolution magnified image, which is taken by the long focus (*f* = 81 mm) of the BDL, and its resolution is up to 21.90″. The other one is the wide FOV (~ 12°) with long DOF (~ 200 μm) image, which is taken by the short focus (*f* = 27 mm) of the BDL, and two objects spread 2880 mm apart can be imaged clearly by the short focus. Thus, a high-resolution magnified image and a wide FOV with long DOF image are taken by the BDL with two specific designed foci, simultaneously. The proposed BDL under the proposed design approach is very suitable to be used in a dual-sensor imaging system, which makes it more integrated and miniaturized to thereby reduce weight, cost, and associated complexity.

## Data Availability

All data generated or analyzed during this study are included in this published article (and its supplementary information files).
